# Which Obstacles Prevent Us from Recruiting into Clinical Trials: A Survey about the Environment for Clinical Studies at a German University Hospital in a Comprehensive Cancer Center

**DOI:** 10.3389/fonc.2017.00181

**Published:** 2017-08-28

**Authors:** Christoph Straube, Peter Herschbach, Stephanie E. Combs

**Affiliations:** ^1^Department of Radiation Oncology, Klinikum rechts der Isar, Technische Universität München (TUM), Munich, Germany; ^2^Deutsches Konsortium für Translationale Krebsforschung (DKTK) – Partner Site München, Munich, Germany; ^3^Roman Herzog Comprehensive Cancer Center (RHCCC), Munich, Germany; ^4^Klinik für psychosomatische Medizin und Psychotherapie, Klinikum rechts der Isar, Technische Universität München (TUM), Munich, Germany; ^5^Department of Radiation Sciences (DRS), Institute for Innovative Radiotherapy (iRT), Helmholtz Zentrum München, Oberschleißheim, Germany

**Keywords:** barriers to participation, clinical trials as topic, survey, physicians, management

## Abstract

**Background:**

Prospective clinical studies are the most important tool in modern medicine. The standard in good clinical practice in clinical trials has constantly improved leading to more sophisticated protocols. Moreover, translational questions are increasingly addressed in clinical trials. Such trials must follow elaborate rules and regulations. This is accompanied by a significant increase in documentation issues which require substantial manpower. Furthermore, university-based clinical centers are interested in increasing the amount of patients treated within clinical trials, and this number has evolved to be a key quality criterion. The present study was initiated to elucidate the obstacles that limit clinical scientists in screening and recruiting for clinical trials.

**Methods:**

A specific questionnaire with 28 questions was developed focusing on all aspects of clinical trial design as well as trial management. This included questions on organizational issues, medical topics as well as potential patients’ preferences and physician’s goals. The questionnaire was established to collect data anonymously on a web-based platform. The survey was conducted within the Klinikum rechts der Isar, Faculty of Medicine, Technical University of Munich; physicians of all levels (Department Chairs, attending physicians, residents, as well as study nurses, and other study-related staff) were addressed. The answers were analyzed using the Survio analyzing tool (http://www.survio.com/de/).

**Results:**

We collected 42 complete sets of answers; in total 28 physicians, 11 study nurses, and 3 persons with positions in administration answered our survey. The study centers reported to participate in a range of 3–160 clinical trials with a recruitment rate of 1–80%. Main obstacles were determined: 31/42 (74%) complained about limited human resources and 22/42 (52%) reported to have a lack on technical resources, too. 30/42 (71%) consented to the answer, that the documentation effort of clinical trials is too large. A possible increase of the patients’ study participation rate up to over 20% was deemed to be possible if the described limitations could be overcome.

**Discussion:**

The increasing documentation effort in clinical trials has led to a strong increase in the work load of scientific personnel. Recruiting of patients into clinical trials therefore is not only limited by patient issues, but also by the infrastructure of the centers. Especially the lack of study nurses is likely to be a major limitation. Furthermore, technical resources for time efficient and safe documentation within clinical routine as well as in clinical trials are required. By optimization of these factors, a significant increase in the amount of patients treated in clinical trials seems to be possible.

## Introduction

Prospective clinical trials, especially randomized controlled trials, are accepted as the most important source of evidence in most subdisciplines of modern medicine; data from clinical trials do constantly shape our guidelines for clinical practice and thus contribute essentially to up-to-date patient care (1, 2). However, gaining this evidence is often hampered by low patient accrual-rates that subsequently can lead to a failure of important trials because they do not reach the preplanned sample sizes in adequate time intervals (3). Many studies have been conducted to investigate the role of patients in this recruitment dilemma. It has been shown that, besides other factors, patients’ concerns about the possibility to be randomized to a placebo treatment or to the standard treatment arm can lead to the hesitation to give informed consent to participate in a trial (4). Also the consent process itself has been identified as one barrier for patients as well as for clinicians to participate in clinical trials (5).

Focusing only on the unwillingness of patients to participate in clinical trials, however, might be an oversimplification of this problem. Trial recruitment depends on several factors, which can also be organizational, financial, or related to other factors relevant to trial management. Therefore, mainly focusing on the patient itself may underestimate the difficulties. Surprisingly, these factors have only been studied marginally so far, although time shortenings and lack of study staff have been blamed as barriers for clinical trials (5, 6).

In order to optimize the recruitment of patients into clinical trials in our hospital, we conducted informative interviews, generated and conducted a quantitative survey, and discussed the results at an open conference at our center. We hypothesized, that recruitment into clinical trials is also limited by infrastructural shortenings. This was confirmed by the responses to our questionnaire. The quantitative results of this survey gave us strong arguments to ease financial resources and to force changes in the administrative framework. We hereby present the results from our survey as similar obstacles might be present in other clinical trial centers, too.

## Materials and Methods

### Questionnaire Development

After a series of informative interviews with department chairs and leading senior professionals from 17 departments of the University Hospital Klinikum rechts der Isar, Technical University of Munich (TUM), Germany, a specific questionnaire was developed, focusing on all aspects of clinical trial design as well as trial management. The questionnaire included 28 questions. We included demographic questions, namely two multiple choice questions about the position in the clinic and the predominant medical field (i.e., medical vs. surgical treatments), two dichotomous questions about the sex of the participant and whether the participant belongs to the TUM and seven open-ended questions asking for the age of the participant, the years of experience in clinical trials, the number of clinical trials and study nurses within the clinical center, and the amount of patients that are treated within different subtypes of clinical trials. Subsequently, 16 rating-scale questions asked whether patient relate complaints, trial factors, structural aspects, and complaints or administrative obstacles had an influence onto the rate of patients recruited to clinical trials (Table 1). Lastly, an open-ended questions asked to which extent the amount of patients within clinical trials could be increased if the obstacles stated in the rating-part would be eliminated. Additionally, the responders had the possibility to leave comments at the end of the survey.

**Table 1:** Questions and results from the questionnaire (translated from German language).

	“I do agree totally” or “[…] mostly”			*p*-Values
	All	Physicians	Study nurses	
**Trial related**				
There are currently no trials available for our patient cohort	2 (4%)	2 (7%)	0	0.351
Important trials could not be established at our center	13 (29%)	12 (41%)	1 (8%)	0.039
The inclusion and exclusion criteria are to exclusive	4 (9%)	3 (10%)	1 (8%)	0.843
Concerning the conduction of clinical trials, I am discouraged by legal regulations (German medical law, German medical technology law, German law for radiation protection, etc.)	10 (23%)	10 (34%)	0	0.019
We offer several trials with almost identical inclusion and exclusion criteria (that do compete to each other)	11 (25%)	8 (28%)	3 (25%)	0.865
**Patient related**				
Most patients do refuse to participate in clinical trials	4 (9%)	1 (3%)	2 (17%)	0.139
I want to protect my patient from additional stress that could be caused by participating in clinical trials	0	0	0	n/a
**Organization related**				
The documentation effort of clinical trials is to large	32 (73%)	20 (69%)	10 (83%)	0.345
The documentation effort within the daily clinical routine hampers me to recruit patients to clinical trials	22 (50%)	14 (48%)	7 (58%)	0.558
There are not enough human resources to conduct (more) clinical trials	31 (70%)	20 (69%)	8 (67%)	0.886
Technical resources or software solutions for the conduction of clinical trials are lacking	23 (52%)	15 (52%)	7 (58%)	0.699
Structural shortcomings (technical and/or human resources) already resulted in a refusal from sponsors to initiate a trial at our center	5 (11%)	4 (14%)	1 (8%)	0.627
Within the last years, trials could not be initiated due to administrative hurdles	10 (23%)	7 (24%)	2 (17%)	0.599
We do not have enough collaborators to conduct clinical trials	5 (11%)	4 (14%)	0	0.176
Conflicts within our center do negatively interfere with our recruiting activity	6 (14%)	3 (10%)	2 (17%)	0.574
I am not experienced enough yet to conduct clinical trials	2 (4%)	1 (3%)	1 (8%)	0.509

*The results represent the complete or predominant agreement to the given statements. The frequency of the answers from physicians and study nurses were compared with the χ^2^ test*.

Furthermore, questions on the medical background and the organizational structure of the participant’s centers were included. The questionnaire was piloted within a small cohort of physicians. Participants of the pilot run were instructed not to participate in the final run of the survey, however, as we performed an anonymous web-based survey it cannot been ruled out that some participants from the pilot run also answered the final questionnaire.

The questionnaire was established to collect data anonymously on a web-based platform (the entire questionnaire in German language can be found within Table S1 in Supplementary Material). The survey was conducted within the Klinikum rechts der Isar, Faculty of Medicine, TUM; clinical scientists of all levels (Departments Chairs, attending physicians, residents, as well as study nurses, and other study-related staff) were addressed. The questionnaire was performed within the quality assurance program of the Comprehensive Cancer Center of our hospital and was therefore in line with the institutional guidelines of the local Ethic’s committee. The answers were analyzed using the web-based Survio analyzing tool (http://www.survio.com/de/) and Microsoft© Excel 2016. The frequency of the answers given by physicians and study nurses were compared with each other using the χ^2^-test function of SPSS v. 18 (IBM).

## Results

The web-based survey counted 120 visits resulting in 44 completed questionnaires (37%). Twenty-nine physicians, most of them senior physicians (20), 12 study nurses, and 3 persons with administrative areas of responsibility completed the questionnaire. Eighteen physicians were from non-surgical and medical specialties, eight physicians had a surgical background, and three physicians had a surgical as well as a medical background. All physicians reported to have long-term experiences in conducting prospective medical trials (median 10 years, range 4–27 years).

Study nurses were employed in surgical disciplines in one (8%) case, in medical disciplines (e.g., internal medicine or radiation oncology) in seven cases (58%), and in subjects with medical and surgical treatments in four cases (33%). The level of experience was comparable with the group of physicians (median 10 years, range 1–15 years). Based on the composition of this cohort, the sample has to be considered as a random sample, as especially department chairs and residents are underrepresented.

The participants did also answer questions about their study centers. Overall, the centers reported to have 0–160 active trials (median 15 trials). Compared with the large number of trials, centers employed only a relative small number of study nurses (median 3, range 0–6), indicating that this might limit the maximum number of patients recruited into clinical trials; in average, every study nurse cared for 10.5 clinical trials. 32 of 44 persons (73%, 7 totally agreed, 25 agreed mostly, Table 1) consented to the answer, that the documentation effort of clinical trials is to large (10 of 12 study nurses, 20 of 29 physicians). Focusing on the clinical routine, 14 physicians (48%) affirmed the statement, that a large burden of documentation is an important obstacle in recruiting patients to clinical trials. Deficits in information technology resources were described by 52% of the responders.

Consistent to this findings, limited human resources were complained by 31 persons (70%; 19 agreed totally, 12 agreed mostly). Additionally, eight responders highlighted this topic within the free-text answers. Limited resources in information technology were also reported by the participants, although by a smaller number (23 of 44 answers, 52%).

Trial-related factors as well as patient-related factors were deemed to have less influence on the recruitment rates, only two physicians (7%) answered, that there are not enough trials available for patients treated at their department. Also refusal from patients to participate in prospective clinical trials seems to be a minor issue in our center (one physician agreed mostly onto that statement).

Altogether, obstacles in infrastructure as well as limitation of human resources were deemed to limit the number of recruited patients by all but six participants. Vice versa, the participants expected to be able to substantially increase the recruitment of patients to clinical trials in 39 cases (89%) if the obstacle would be improved.

We grouped the answers of the responders according to their job type (e.g., physicians vs. study nurses) and compared the frequency of the answers. There were no significant differences in the frequency of affirmations to questions asking for patient- or organization-related factors. However, physicians agreed significantly more to the statements “Important trials could not be established at our center” (*p* = 0.039) and “Concerning the conduction of clinical trials, I am discouraged by legal regulations […]” (*p* = 0.019).

## Discussion

In the present analysis, we sought to determine major obstacles for prospective clinical trials. In a detailed questionnaire, we identified documentation tasks as a key factor, as well as the difficulty to recruit well trained staff. Limited personnel resources may be a main obstacle for effective conduction of clinical trials. Furthermore, the majority of participants suspected an increased efficacy in patient recruiting if these obstacles would be eased.

Clinical trials are the most important sources for valuable evidence in modern medicine. Unfortunately, a large proportion of trials undergo early closure due to poor accrual. While patient-related obstacles for the recruitment into clinical trials have already been studied, literature on institutional obstacles for trial recruitment is scarce. A large documentation due to rising quality claims and increasing legal regulations are reasons for the growing documentation effort in clinical trials that leads to an increase of workload for the scientific personnel in university hospitals (6–8). Besides there is no evidence that this continuous increase in regulation efforts does really improves the quality of scientific results, conduction of clinical trials is increasingly complex. Consequently, the vast majority of our participants did agree to the statement, that the documentation effort of clinical trials is to large. As many responders complained about large documentation efforts within the routine treatment of patients and about deficits in the information technology infrastructure, one could summarize that infrastructural shortenings do currently limit time of physicians and study nurses to an extent that precludes the recruitment of more patients. While this already seems to be a significant problem at a German university hospital, centers within the developing world do suffer even more from this development (6, 7). Therefore, the discussion of this topic should be continued although the recently updated GCP guidelines do allow “more efficient approaches to clinical trial design […], recording [… and] reporting” (9). It is within the responsibility of future sponsors and investigators to optimize their protocols to consequently reduce the requested information to the least necessary amount.

Current trials, however, still suffer from the large burden of necessary documentation which only can be handled by the help of an adequate number of supportive personal (5). This was confirmed by one key-finding from our survey: a shortening of human resources is one of the most important obstacles for increasing the rate of patients recruited to clinical trials. This was also reported within a survey by Kaanoi et al., who reported that 22 of 27 oncologists in Hawaii did not have enough support staff to recruit more patients to clinical trials; he explained, that the low number of study nurses limited the amount of patients in clinical trials for which all quality claims for clinical trials could be fulfilled (10). Furthermore, a systematic review by Fisher and colleagues summarized, that a lack of time for the screening, treatment, and follow up of clinical trials is a major barrier for oncologists to participate in clinical trials (5). Notably, the increasing documentation duties within the clinical routine leads to a further increase of this barrier, a finding that was already described in the early 1990s in the United Kingdom (11). While especially investigator initiated trials are often underfinanced, a political debate on the necessity of supportive scientific personnel in clinical centers is needed. Furthermore, the additional personnel effort should be taken into consideration when contracts for company initiated trials are made. Concerted lists for the costs of study personal as well as medial measures, as already common for the pharmacists in dispensaries in Germany, could become an important tool for the planning, contracting, and the conduction of clinical trials.

The results of our survey cannot be generalized to the level of individuals of our center, as especially department chairs, and residents are underrepresented. However, the survey was answered mostly by experienced physicians involved in the conception of clinical trials as well as in the management of the scientific centers. The second largest group consisted of the supportive scientific staff. These two large groups are likely to give valuable information about their centers since they are the two main groups in charge of day-to-day issues in clinical trial management. Therefore, the obstacles reported by the participants are likely to represent the most important institutional barriers for patient recruitment to clinical trial at our center. Whether the results of our survey can be generalized to other centers can hardly be answered, as key values needed for a comparison, i.e., the number of study nurses or the number of active trials, are not available for other centers. However, since at least within Germany University Hospitals are characterized by similar organizational structures most arguments most likely hold true for other sites. Further investigations about an ideal balance between the number and complexity of clinical trials at one center, the number of study nurses and the number of patients within clinical trials are therefore highly recommended.

Until that, a continuous review of the study process on all levels of a scientific clinical center seems to be a sufficient tool to identify barriers for the conduction of clinical trials. Of importance, patient related as well as structural factors need to be analyzed to improve the process of clinical trials. Results of quantitative surveys can help to hierarchical sort the importance of administrative hurtles and can serve as arguments for easing financial of personnel resources for their solution. Coming back to our experiences, the results from the interviews as well as from the survey allowed us to build an interdisciplinary consent about the most important issues, and some of the most important issues have already been solved. Additionally, clinical scientists should take the personal limitations of clinical centers into account when new protocols for clinical trials are generated. A lower burden of documentation, partially by focusing onto the main objectives of the trial, can help to increase the efficacy of the trial centers which subsequently can handle more patients within clinical trials.

## Author Contributions

CS drafted and designed the survey, approved the survey, analyzed the results, and wrote the manuscript. PH and SC designed the survey, approved the survey, critically discussed the results, gave important intellectual input, and wrote the manuscript. All authors approved the final version for the manuscript.

## Conflict of Interest Statement

All authors declare to have neither financial, commercial nor any author conflict of interest that could affect the results or the discussion of the content of the manuscript.
