# Supplementary material for: Which Obstacles Prevent Us from Recruiting into Clinical Trials: A Survey about the Environment for Clinical Studies at a German University Hospital in a Comprehensive Cancer Center
Source: Front Oncol. 2017 Aug 28;7:181. doi: 10.3389/fonc.2017.00181 (PMC5581318; doi:10.3389/fonc.2017.00181)
Supplement: Supplementary file 1 [file table_1.pdf]

Anhang: Umfrage

Umfrage zur Studientätigkeit am CCC München

Sehr geehrte Kollegen,

Diese Umfrage soll helfen, Probleme bei der Initiierung und Durchführung klinischer Forschung an unseren Kliniken zu erfassen um diese Probleme danach strukturiert angehen zu können. Um möglichst fundierte Aussagen bekommen zu können ist es wichtig, dass möglichst viele Mitarbeiter aus den Zentren an der Umfrage teilnehmen. Konkret richtet sich diese Umfrage sowohl an die ärztlichen Mitarbeiter als auch an die Studienkoordinatoren und Studienschwestern. Selbstverständlich ist diese Umfrage streng vertraulich, die Daten lassen keinen Rückschluss auf Ihre Person oder ihre genaue Tätigkeit zu.

Für Ihre Zeit möchten wir uns herzlich bedanken,

Ihr Dr. Christoph Straube und Dr. Lars Schüttrumpf Koordinatioren für klinische Forschung am CCC München

In welcher Position sind Sie mit Studien betraut?

- ○ Chefärztin / Chefarzt
- ○ Oberärztin / Oberarzt
- ○ Fachärztin / Facharzt
- ○ Assistänzärztin / Assistenzarzt
- ○ Studienkoordinator / Studynurse
- ○ Organisatorisch / Verwaltung / Apotheke / Infrastruktur

Welches Geschlecht haben Sie?

- ○ Weiblich
- ○ Männlich

Wie alt sind Sie?

An welchem Standort sind Sie beschäftigt?

- ○ Klinikum rechts der Isar / TUM
- ○ Klinikum der Universität München / LMU

Ihre Tätigkeit ist:

- ◯ vorwiegend operativ
- ◯ vorwiegend konservativ
- ◯ operativ und konservativ, etwa gleichberechtigt

Über wieviel Jahre Erfahrung verfügen Sie bei der Durchführung klinischer Studien?

Wieviele klinische Studien (mit Ethikvotum und prospektiv erfasstem Endpunkt, z.B. Studien nach AMG, MPG, Anwendungsbeobachtungen, QoL, Biomarker, Supportivmedizin etc.) werden in Ihrer Klinik durchgeführt?

Wieviele Studienkoordinatoren / Studynnurses arbeiten in Ihrer Klinik?

Wieviel Prozent der erwachsenen Patienten mit soliden Tumoren nehmen in Ihrer Klinik an klinischen Studien (inkl. Biomarker, Supportive Care, QoL etc.) teil? (wenn nicht zutreffend dann bitte überspringen)

Wieviel Prozent der erwachsenen Patienten mit hämatologischen Malignomen nehmen in Ihrer Klinik an klinischen Studien (inkl. Biomarker, Supportive Care, QoL etc.) teil? (wenn nicht zutreffend dann bitte überspringen)

Wieviel Prozent der pädiatrischen Patienten mit Malignomen nehmen in Ihrer Klinik an klinischen Studien (inkl. Biomarker, Supportive Care, QoL etc.) teil? (wenn nicht zutreffend dann bitte überspringen)

Bitte bewerten Sie folgende Aussagen für Ihr persönliches Arbeitsumfeld.

	Trifft voll zu	Trifft eher zu	Trifft eher nicht zu	Trifft überhaupt nicht zu	N/A
Für unser Patientenkollektiv gibt es aktuell keine relevanten Studienansätze.	○	○	○	○	○
Wichtige Studien konnten bisher nicht an unser Zentrum geholt werden.	○	○	○	○	○
Wir verfügen über mehrere Studien mit nahezu identischen Ein- und Ausschlusskriterien.	○	○	○	○	○
Die Ein- und Ausschlusskriterien der bei uns verfügbaren Studien sind zu kleingliedrig.	○	○	○	○	○
Klinische Studien werden von den meisten Patienten ablehnend bewertet.	○	○	○	○	○
Ich möchte meinen Patienten die Belastung klinischer Studien ersparen.	○	○	○	○	○
Der Dokumentationsaufwand für Studien ist zu hoch.	○	○	○	○	○
Der Dokumentationsaufwand (Doppeldokumentation) in der klinischen Routine hält mich von der Studienarbeit ab.	○	○	○	○	○
Es fehlt an personellen Ressourcen für die Durchführung von Studien.	○	○	○	○	○
Es fehlt an technischen / informationstechnologischen Ressourcen für die Durchführung von Studien.	○	○	○	○	○
Industriegeförderte Studien wurden in der Vergangenheit bereits mit Verweis auf die Infrastruktur, die technischen oder personellen Ressourcen vom Sponsor abgelehnt.	○	○	○	○	○
Studienvorhaben wurden in der Vergangenheit bereits an administrativen Hürde gestoppt (Rechtsabteilung, Ethik, etc.).	○	○	○	○	○
Die rechtlichen Reglementierungen (AMG, MPG, Strahlenschutz, Archivierung etc.) halten mich von Studien ab.	○	○	○	○	○
Für die Durchführung von klinischen Studien fehlen uns aktuell Kooperationspartner in anderen Abteilungen.	○	○	○	○	○
In unserem Zentrum gibt es Zerwürfnisse die die Rekrutierung von Patienten für Studien behindern.	○	○	○	○	○
Mir fehlt die Erfahrung für die Durchführung einer klinischen Studie	○	○	○	○	○

Wenn die oben genannten Probleme beseitigt wären, um wieviel Prozent könnten Sie (realistisch) die Rekrutierung von Patienten für klinische Studien steigern?

Haben Sie noch Anmerkungen, Anregungen, Bitten, Kommentare?
